# Screen-Printed Gold Electrodes as Passive Samplers and Voltammetric Platforms for the Determination of Gaseous Elemental Mercury

**DOI:** 10.1021/acs.analchem.0c04347

**Published:** 2021-02-01

**Authors:** Samuel Frutos-Puerto, Conrado Miró, Eduardo Pinilla-Gil

**Affiliations:** † Department of Analytical Chemistry, University of Extremadura, Av. de Elvas, s/n, 06006 Badajoz, Spain; ‡ Department of Applied Physics, University of Extremadura, Av. de la Universidad, s/n, 10005 Cáceres, Spain

## Abstract

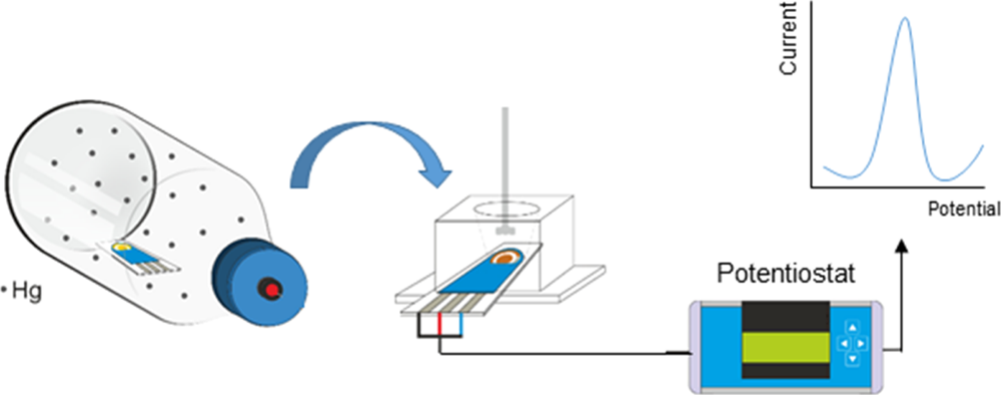

We present a methodology for the determination of gaseous elemental mercury (GEM). It is based on passive sampling of Hg on screen-printed gold electrodes (SPGEs), followed by the measurement of amalgamated mercury by square wave anodic stripping voltammetry. We have explored in detail the behavior of the SPGE electrode surface during the sampling process (by time-of-flight secondary ion mass spectrometry), the stability of the voltammetric signals, and the inter-electrode reproducibility, and obtained acceptable results. Adsorption of mercury onto the SPGE follows a nearly linear behavior until the sorbent becomes saturated (equilibrium phase) for different mercury concentrations, allowing to select a sampling time of 30 min for calibration. The theoretical behavior of the sampling system was modeled, considering the changes in the diffusive path length between the porous diffusive barrier and the adsorbed surface, *L*. Finally, we have tested two GEM calibration protocols. The first one is based on the measurement of the mercury stripping peak area, *A*_Hg_, and the second one is based on the measurement of the mass of mercury, *m*_Hg_, by standard additions. We found good correlation coefficients between the GEM concentration for both *A*_Hg_ (*R*^2^ = 0.9591) and *m*_Hg_ (*R*^2^ = 9615) in the range of 5.82 to 59.29 ng dm^–3^ GEM. Detection limits were 5.32 and 5.22 ng dm^–3^ for *A*_Hg_ and *m*_Hg_, respectively. Our results open a new line of electroanalytical strategies for the determination of GEM in atmospheric samples.

## Introduction

Gaseous elemental mercury (GEM) is the dominant Hg species in the atmosphere (>90%)^1^ and has the potential to be deposited at 0.01 cm s^–1^^2^ on soil or aquatic environments, and consequently, to be converted to other toxic inorganic and organic forms.^3^ These forms of Hg(II) may be consumed by humans through drinking water or food harming their health.^4^ Besides, GEM can be directly inhaled and absorbed through the respiratory tract.^5^ The most significant sources of mercury emission into air are point sources such as industrial facilities and diffuse sources such as internal combustion engines in a large city.^6−8^ Once in the atmosphere, elemental mercury can disperse for long distances,^1,9,10^ remaining in air for up to 2 years.^1^ These reasons justify the well-established regulations about monitoring and reducing ambient mercury concentrations.^11^ The World Health Organization (WHO) has set up a guideline of 1 ng dm^–3^ for inorganic mercury vapor as an annual average.^12^ Considering that typical levels of mercury in outdoor air are in the range of 0.005–0.010 ng dm^–3^, it becomes evident that there is a need for sensitive, selective, fast, and decentralized methods and devices for efficient control of GEM levels. The available methodologies start with the active or passive collection of Hg^0^ from air, gold being considered a standard accumulation medium due to its unique property to form a gold–mercury amalgam.^13^ The use of gold or modified gold as a sorbent material of passive air samplers (PASs) has been described in the literature.^14−18^ These studies explore different geometries for the sampling box where the adsorption of mercury over the sorbent is governed by turbulent and molecular diffusion, so the amount of target mercury that is taken up by the sampler from the surrounding air over a given period can be quantified.

The GEM collection or pre-concentration step is followed by the measurement of an analytical signal, based on either the observation in the change in the properties of the gold film^19^ or by thermal desorption and subsequent quantification of mercury by techniques such as cold vapor atomic fluorescence or atomic absorption spectroscopy.^20^ These techniques are specific, reliable, and have excellent sensitivity; however, they have notable disadvantages such as high cost, in situ analysis limitations, and the need for highly skilled technicians. Voltammetric techniques have proven to be a valid, miniaturized, and low-cost alternative for mercury detection, applicable for decentralized analysis. Specifically, screen-printed electrodes (SPEs) are widely used for the voltammetric determination of heavy metals,^21,22^ including mercury,^23,24^ in various applications. Nevertheless, methods reported in the literature for voltammetric analysis focus on the soluble species Hg(II), most of them employing gold electrodes, including solid gold disk or gold film electrodes^25−28^ or screen-printed gold electrodes, SPGEs.^29−32^

Typical SPGEs that are commercially available are cured at high (HT-SPGE) or low (LT-SPGE) temperatures. The main difference between both electrodes is the narrower potential working range of HT-SPGEs,^29^ and the need for an activation process for Hg(II) signal enhancement,^26−28,33,34^ both of them being suitable for Hg(II) detection.

No reports have been found in the literature about the use of SPGEs as a PAS for GEM collection and the subsequent voltammetric determination. However, the overall methodological approach was first described by Scholz et al.^35,36^ for the ASV measurement of dissolved Hg(II) after reduction to Hg(0) with Sn(II) and sorption of the volatilized GEM on a rotating gold disk electrode, who proposed the sorption from a gas phase as a new pre-concentration method in stripping voltammetry.^37^ The same authors have described in detail the standard potentials of the redox electrodes’ “dissolved atomic mercury/dissolved mercury ions”,^38^ the thermodynamic effects derived from the presence of atomic mercury at low concentration levels,^39^ and the speciation of mercury for dissolved atomic mercury, dissolved ionic mercury, and total mercury.^40,41^ The adsorption mechanism of GEM on gold thin-film substrates depends on the temperature and exposure time^13^ and tends to exhibit a saturation level. For this reason, it is critically important to know the evolution of the amount of amalgamated mercury as a function of time in the PAS to obtain the relation between the GEM concentration in ambient air and the mass of mercury accumulated.

The aim of this work was the development and preliminary laboratory testing of a novel method for the determination of GEM in ambient air, employing commercial SPGEs as passive sampling and detection devices. After passively collecting the analyte from air by amalgamation on the SPGE, we employed square wave anodic stripping voltammetry (SWASV) to strip amalgamated mercury from the surface, measuring the resultant current.

## Materials and Methods

### Chemicals and Reagents

Mercury metal (Panreac, Spain) was the source of GEM inside the “bell-jar” apparatus (see the Experimental Setup and Procedure for Gaseous Mercury Standards Generation). The Hg(II) standard stock solution (10 mg L^–1^, ICP quality) employed for SWASV measurements was from PerkinElmer (Spain) and diluted as required. All solutions were prepared from ultra-pure water (18.2 MΩ cm) obtained from a Wasserlab Ultramatic system (Navarra de Tratamiento de Agua S.L., Pamplona, Spain). Hyperpure grade HCl, supplied by Panreac (Spain), was used to prepare a 10^–1^ M solution for adjusting the samples to pH 1. All the materials used were washed adequately by immersion in a 10% sub-boiled HNO_3_ solution for 1 week. The sub-boiled HNO_3_ was obtained from a quartz sub-boiling system (Kürner, Rosenheim, Germany).

### Apparatus

**Figure 1:**
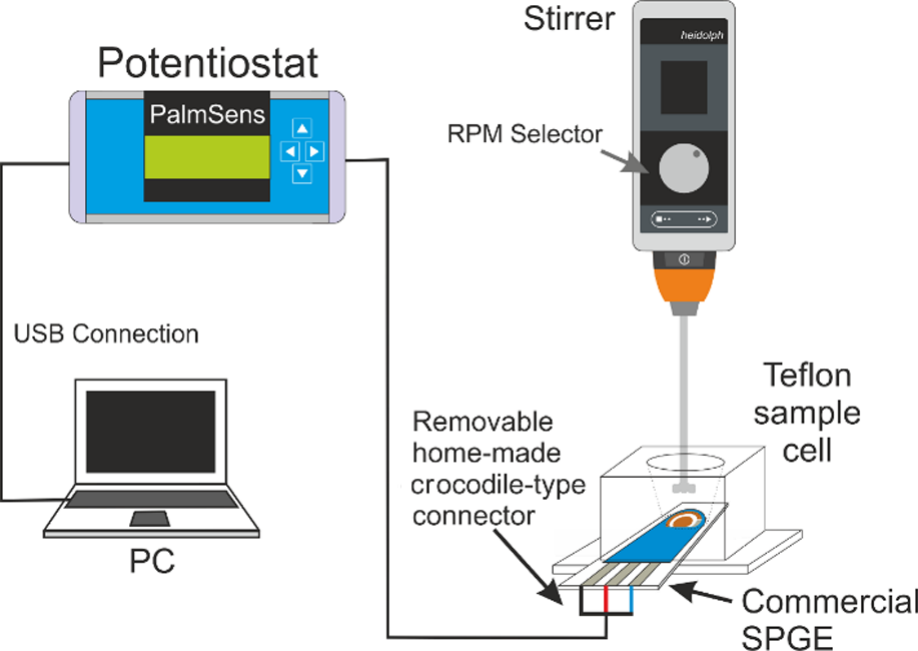
Experimental setup for the determination of Hg(II) by SWASV on SPGE.

A PalmSens2 potentiostat/galvanostat (Palm Instruments BV, The Netherlands), controlled by PSTrace v.5.6 software, was used for voltammetric measurements. The experimental setup also includes a precise (1 rpm resolution) manually controlled stirrer (Heidolph Schwabach, Germany) in the 0–2000 rpm range and a Teflon-made customized cell for SPEs, model CFLWCL-CONIC, from Methrom-DropSens (Oviedo, Spain). The capacity of this novel cell is up to 2.0 mL sample solution for batch analysis, allowing convenient overhead stirring and spiking of the solution to perform standard addition methods. Methrom-DropSens provided disposable LT-SPGEs (ref. 220BT), consisting of a working electrode (sputtered thin gold film of 4 mm diameter), a counter electrode (same material as the working electrode), and a silver pseudoreference electrode, printed on a ceramic surface. The electrodes were firmly connected to the potentiostat through a hand-modified crocodile-connector wire, in replacement of the original sliding connector that we have observed to be very prone to unexpected disconnections (Figure 1). For surface morphology characterization, we used a scanning electron microscope FE-SEM Quanta 3D FEG (FEI Company, Oregon, EE.UU.). Qualitative microanalysis of Au–Hg amalgam by time-of-flight secondary ion mass spectrometry (TOF-SIMS) was carried out using a TOF-SIMS^5^ instrument (IONTOF, Munster, Germany).

### Experimental Setup and Procedure for Gaseous Mercury Standard Generation

**Figure 2:**
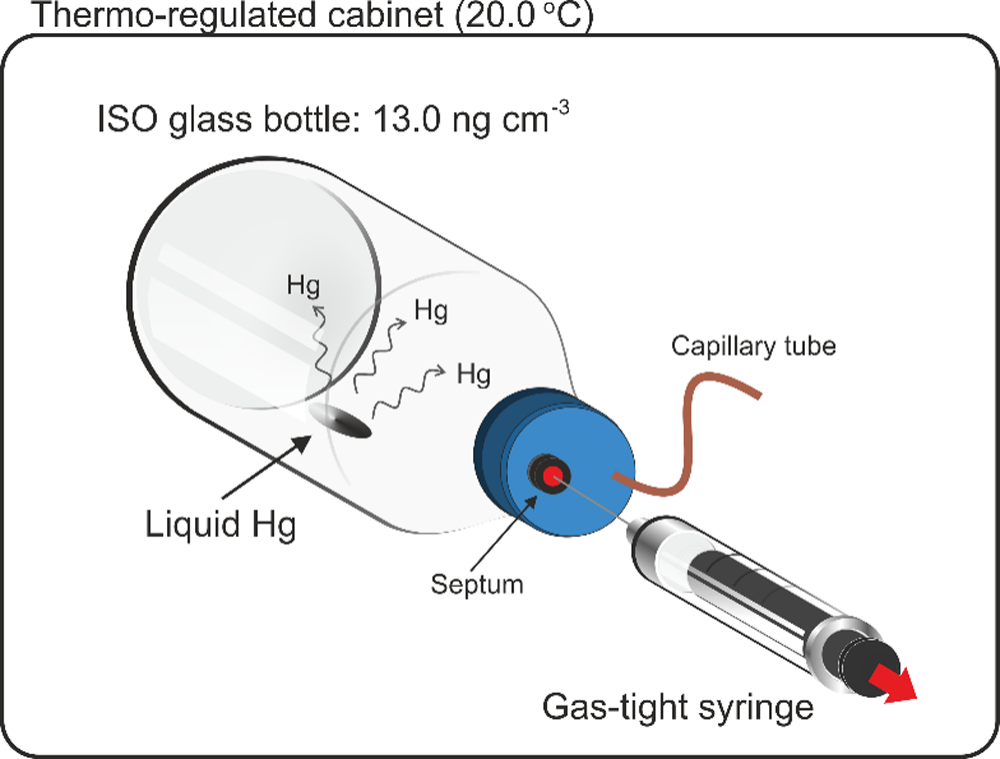
Experimental setup for gaseous mercury standard generation. Mother vapor contains 13.0 ng cm^–3^ gaseous mercury under dynamic equilibrium.

The device used for generating standard concentrations of GEM was a “bell-jar”^42,43^ commonly used to calibrate mercury detection instruments. A small amount of liquid mercury establishes a dynamic equilibrium with the surrounding atmosphere, generating an elemental mercury-saturated atmosphere inside the jar whose concentration can be known as a function of the temperature. Hence, known volumes of mercury can be removed from the jar for calibration purposes. Under experimental conditions similar to those reported by Brown and Brown,^42^ a drop of 16.58 g (2 mm top-view diameter) of liquid mercury was carefully placed onto the base of an ISO borosilicate glass bottle (Scharlab, Spain) with a total volume of 2285.4 mL (GEM stock bottle) placed horizontally (Figure 2). The screw cap of the bottle was modified by adding a small screw cap adapter with a removable septum to allow mercury-saturated air samples to be taken from inside the bottle using a 10 mL gas-tight syringe (SGE Analytical Science, Melbourne, Australia). A capillary tube through the cap equilibrates pressures inside and outside the bottle. The whole system was deployed inside of a thermo-regulated cabinet of approximately 9 m,^3^ at a constant temperature of 20.0 °C, monitored and registered by a Tinitag TV-4500 electronic thermometer (Gemini Data Loggers, Chichester, UK). Under these conditions, the calculated concentration of mercury at dynamic equilibrium was 13.0 ng cm^–3^ according to eq 1(44)
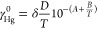
1

Equation 1 describes the model known as the “Dumarey equation”, the most common relationship to estimate the saturated vapor concentration of mercury changes with temperature, where γ_Hg_^0^ is the expected saturated mass concentration of mercury vapor in air (ng cm^–3^), *T* is the temperature of air (K); *D*, *A*, and *B* are constants equal to −8.13 K, 3240.87 K, and 3,216,522.61 K ng cm^–3^, respectively; δ is the deviation of the model from reality, taken as one for zero uncertainty.

### Passive Sampling of GEM on SPGE

**Figure 3:**
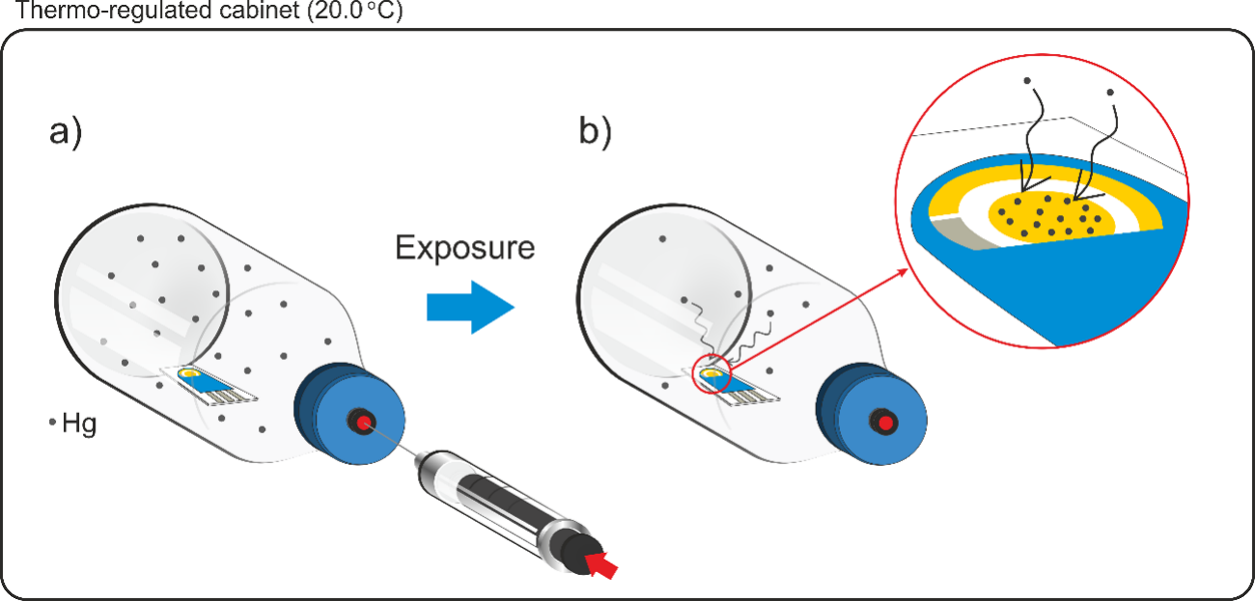
Passive sampling of mercury on the SPGE. (a) Hg(0) atoms are present in air surrounding thee SPGE after being injected into the bottle. (b) Hg(0) amalgamating on the gold surface [Hg(0)Au].

Known volumes of air were taken from the GEM stock bottle and injected into another bottle containing the SPGE as Hg passive samplers (Figure 3a). Under these conditions, the Hg(0) atoms present in air surrounding the electrode interact with the gold-plated surface of the electrode, forming a gold–mercury amalgam (Figure 3b). A temperature-dependent diffusion process governs these interactions.

### Voltammetric Analysis

Two experimental measurement protocols are possible for GEM calibration, as described in detail below.

#### Measurement Protocol 1

**Figure 4:**
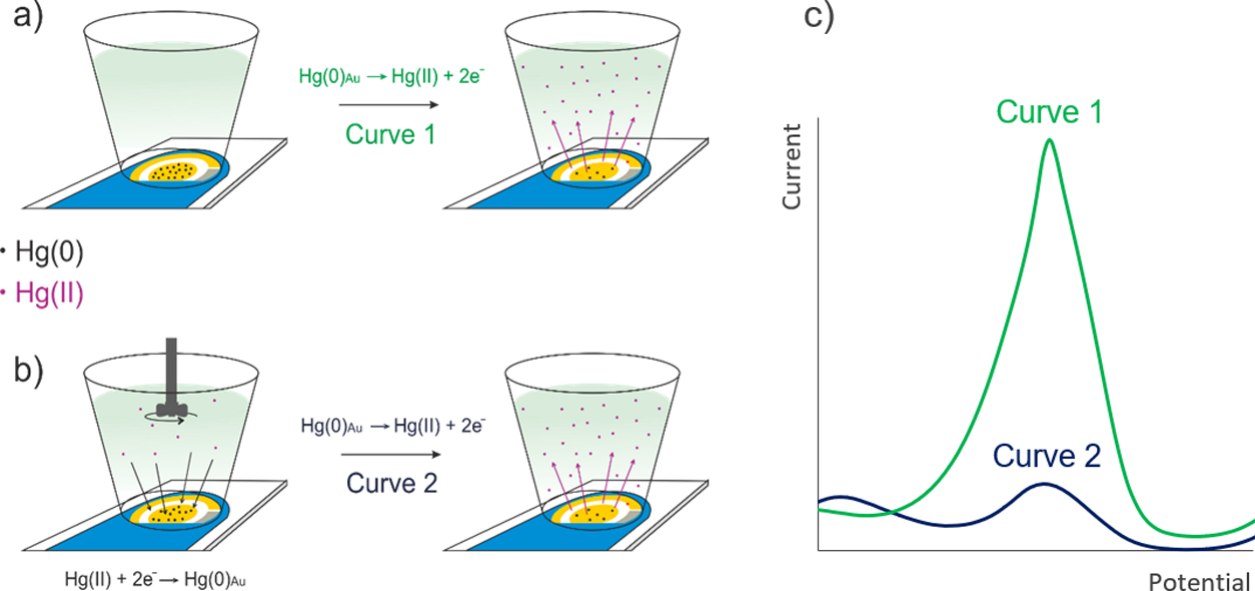
SWASV measurements. (a) Placing the SPGE containing the amalgamated mercury Hg(0)_Au_ in the voltammetric cell, and the first stripping process. (b) Oxidized mercury Hg(II) in the solution is amalgamated and stripped again after the first stripping process. (c) Signals corresponding to the processes (a,b).

According to the schematic experimental setup presented in Figure 1, the SPGE is placed in the measurement cell. 1.5 mL of 0.1 M HCl solution is then added, and the amalgamated Hg(0) atoms are stripped to the solution (Figure 4a) by an anodic sweep (potential sweep from 0.1 to 0.65 V, 6 mV step potential, 40 mV amplitude, and 10 Hz frequency). A high and well-defined peak is obtained (Figure 4c, curve 1), and the peak area *A*_Hg_ is used as the analytical signal.

#### Measurement Protocol 2

After the first measurement described in protocol 1, the concentration of Hg(II) ions in the solution (previously stripped from the SPGE) can be measured by SWASV (Figure 4c, curve 2) under the following experimental conditions: 20 s of conditioning time at 0.7 V, 60 s of deposition time at −0.1 V with 300 rpm stirring rate, 10 s of equilibriation time at −0.1 V, potential sweep ranging from 0.1 to 0.65 V, step potential of 6 mV, amplitude of 40 mV, and frequency of 10 Hz. The working electrode was kept for 10 s at 0.7 V between measurements to clean the surface. This protocol allows the measurement of Hg(II) concentration by standard additions to calculate the mass of GEM trapped by the SPGE during passive sampling, *m*_Hg_.

## Results and Discussion

### SPGE Surface Characterization during Passive Sampling of GEM

We explored in detail the surface of the SPGE working electrode during the passive sampling of GEM by scanning electron microscopy, SEM. Figure S1a shows the more granular and rough structure of LT-SPGE compared to that of HT-SPGE (Figure S1b),^45^ making the LT-SPGE more effective for GEM capture.

Figure S2 shows TOF-SIMS measurements of mercury and gold distribution over the alumina surface after 30 min of exposure to an atmosphere containing a GEM concentration of 56.69 ng dm^–3^. TOF-SIMS was focused on mercury and gold ion extraction from the surface when they are bombarded with bismuth ions (primary ion gun). The surface was cleaned to eliminate surface contamination (mainly from organic molecules adsorbed onto the surface) by applying O_2_ ions at 1 kV of energy and 250 nA of intensity during 3 s, using a raster size much larger than the analysis area. After cleaning, static surface analysis was applied, using Bi^3+^ ions at an energy of 25 kV and an intensity of 0.2 pA under a vacuum of less than 4 × 10^–9^ mbar. This analysis was carried out on an area of 500 × 500 μm with a spectral data collection with an ion dose of 10^12^ ions cm^–2^ and a pulse width of 16.4 ns. Figure S2a shows Au^+^ being homogeneously distributed on the surface as expected. Hg+ and amalgam, AuHg^+^, are present and homogeneously distributed (Figure S2b,c). Figure S2d gives a surface representation where low-intensity zones correspond to holes of the granular structure revealed by SEM.

TOF-SIMS results make it possible to represent the relative amount of mercury amalgamated on the working electrode versus passive sampling time (Figure S3). We took intensity relations coming from the joint spectral signal of the whole area (analyzed areas) to minimize the matrix effects and to give qualitative results about the distribution of elements of interest.

Figure S4 shows the intensities obtained for Hg^+^ ions at times of exposures of 0, 60 and 120 min as a visual representation of the amount of mercury adsorbed at different times. Although the intensities are not relative to the Au^+^ matrix, signal intensities were higher for higher exposure times.

### Stability of Voltammetric Signals

SPEs have delicate surfaces prone to degradation due to the interaction between the working electrode and the media, and the friction suffered during mechanical stirring, so we first explored the behavior of the LT-SPGE used in this study under repeated measurements. Figure S5 shows the results obtained for current peak evolution for a solution of 30 ng mL^–1^ Hg(II) in 0.1 M HCl along 100 measurements on the LT-SPGE under 300 rpm of stirring rate. We choose this rate as a good compromise between high signals and signal stability, although according to Squissato et al.,^32^ higher stirring values are also applicable. RSD was 8% for the 100 measurements and 3% considering the first 50 measurements only. These results prove that LT-SPGEs are suitable for the voltammetric determination of Hg(II) for a higher number of measurements with proper stability. Previous studies carried out under similar experimental conditions on HT-SPGEs and gold nanoparticle-modified screen-printed carbon electrodes (AuNPs-SPCEs)^46^ showed less stable performance with a substantial decrease of approximately 4 times lower than the initial value after 30 measurements. We observed better results for signal stability on AuNPs-SPCEs (without the surface activation process) without stirring during the accumulation time [relative standard deviation (RSD) 6%]. Moreover, as mentioned above, HT-SPGE presents a narrow potential window in comparison with that of LT-SPGE,^29^ and HT-SPGEs need to be activated (several cycles of sweep potential with a proper selection of the supporting electrolyte) to obtain sharp and reproducible signals during voltammetric stripping measurements of Hg(II).^26−28,33,34^

### Inter-electrode Reproducibility

The RSD of a set of three LT-SPGE electrodes after they were exposed for different times to 56.59 ng dm^–3^ GEM was in a range of 1 to 24% (13% average) for the peak area (Measurement Protocol 1 section) and 5 to 9% (6% average) for the mass of Hg adsorbed onto the electrode, *m*_Hg_ (Measurement Protocol 2 section). These repeatability values are acceptable, taking into account the disposable characteristics of these low-cost electrodes. RSD values obtained for *m*_Hg_ show lower errors than *A*_Hg_ which is due to the fact that each value of *m*_Hg_ is obtained as a result of calibration by standard additions.

### Influence of Sampling Time on the Mercury Mass Captured on the SPGE

We measured the value of *m*_Hg_ amalgamated on the SPGE for different GEM concentrations, γ_Hg_^0^, at different sampling times, by taking volumes of 1.0, 5.0, and 10.0 cm^3^ of air from the GEM stock bottle (Figure 2) and injecting them into a second bottle containing the SPGE (Figure 3a). The selected volumes correspond to GEM concentrations of 5.78, 29.40, and 58.31 ng dm^–3^ depending on the recorded temperatures at the time of extraction (20.2, 20.4, and 20.3 °C, respectively). SPGEs were exposed in triplicate for 10, 30, 60, 90, and 120 min, at each GEM concentration value. m_Hg_ was measured by SWASV, as described in measurement protocol 2 (quantification by standard additions).

Figure S6 presents the experimental results of *m*_Hg_ (ng) amalgamated on the SPGE versus sampling time. According to the expected behavior for adsorption onto the PAS of a gaseous substance,^47^ the adsorption of mercury onto the electrode surface follows a nearly linear behavior until the sorbent becomes saturated (equilibrium phase). The same behavior is observed for different GEM concentrations. According to these results, 30 min seems to be a proper compromise for GEM calibration in the studied range.

### Theoretical Description of the Passive Sampler

The theoretical behavior of the SPGE as a PAS for the adsorption of GEM can be described, considering that the diffusive path length between the porous diffusive barrier and the adsorbed surface, *L*, change eventually, unlike a typical PAS.^47^ This length will be set by the diffusion layer caused by the concentration gradient at each time, calculated from the integration of Fick’s second law for a circular flat electrode (eq 2)
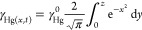
2where *x* is the distance from the electrode surface and *z* is a dimensionless number (eq 3).
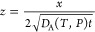
3where *D*_A_ is the molecular diffusion coefficient of mercury in air, given by eq 4,^48^ and *t* is the sampling time.
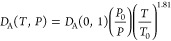
4where *T* [K] and *P* [any appropriate unit] are the temperature and pressure of the system, respectively, *T*_0_ = 273.15 K, *P*_0_ = 1 atm pressure at 0 °C, and *D*_A_(0,1) is the molecular diffusion coefficient at standard temperature and pressure.

**Figure 5:**
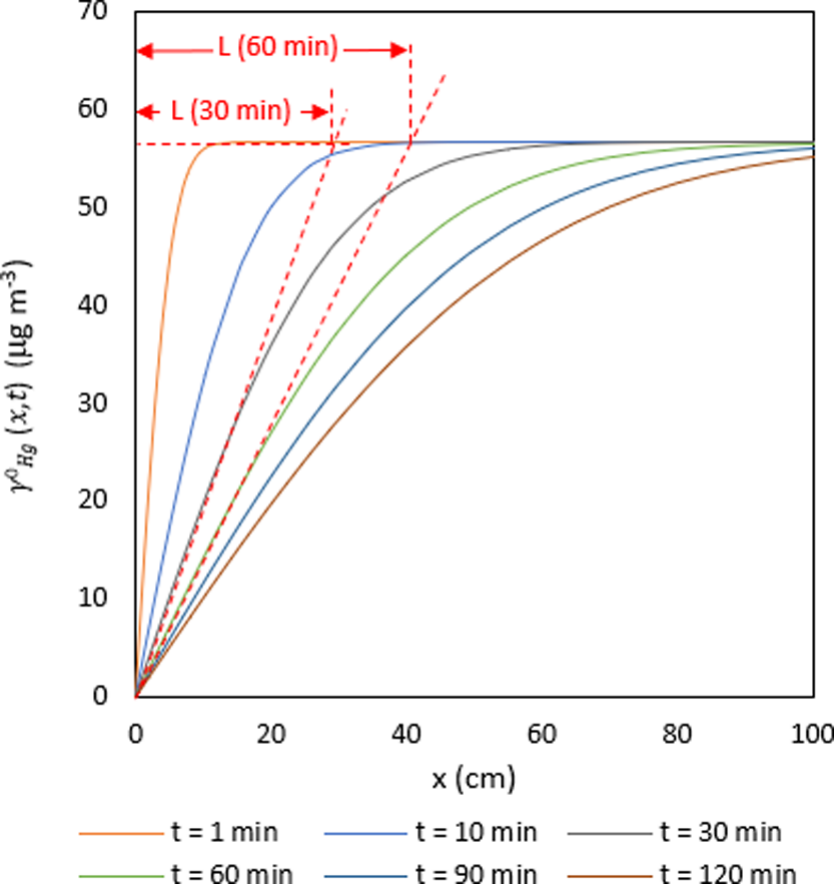
γ_Hg(*x*,*t*)_ profiles for diffusion as a function of time.

Integration of eq 2 gives eq 5, where erf(*z*) is the error function of *z*, which can be represented at different times of exposure giving the values represented in Figure 5.
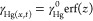
5

As can be seen in Figure 5, *L* can be obtained from the interpolation between γ_Hg_^0^ and the best-adjusted curve at each time. *L* values obtained for each time were 5.46, 16.15, 27.81, 39.28, 48.08, and 55.5 cm for 1, 10, 30, 60, 90, and 120 min, respectively.

Knowing the distance from the electrode for the diffusion layer, we can calculate the uptake rate, UR, from the following equation
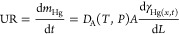
6where A is the area of collection of the PAS and dγ_Hg(*x*,*t*)_/d*L* is the concentration gradient of mercury across *L*, whose integration leads to
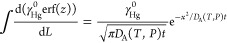
7

Finally, by substituting eq 7 in the UR expression, we can estimate the theoretical amount of mercury adsorbed over the electrode surface at each time of exposure, eq 8
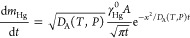
8

**Figure 6:**
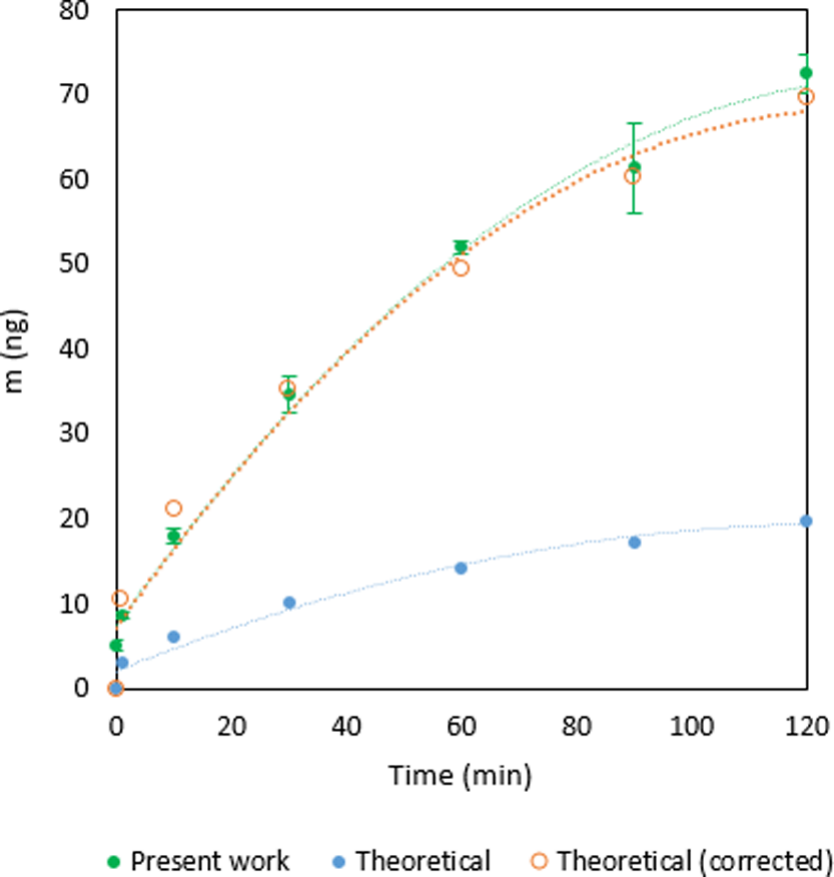
Sorbed *m*_Hg_ theoretical estimation at different times vs experimental values.

The results of *m*_Hg_ for each time theoretically estimated from eq 8 are presented in Figure 6 versus experimental values. The values of *D*_A_(*T*,*P*), *A*, and γ_Hg_^0^ are 0.136 cm^2^ s^–1^, 0.856 cm^2^, and 5.66 ng cm^–3^, respectively.

Figure 6 shows that theoretical values are lower than experimental values. This difference may be explained considering that the experimental value of *A* (geometrical area) is lower than that of the active area, in agreement with the granular microscopic structure shown in Figure S1a. To describe the observed behavior by taking this fact into account, we estimated *A* (active area) to be 3 times higher than the geometrical one (3.0 cm^2^) (whose results are presented in Figure 6). The theoretical behavior is in line with the experimental one for this area.

Apart from UR, another relevant parameter to characterize *m*_Hg_ is the sampling rate SR. For a diffusive PAS, SR quantifies the volume of air that effectively diffuses through the PAS surface per unit time, according to Fick’s first law (eq 9).
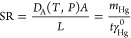
9SRs can be estimated theoretically, but they are usually determined by calibration, using active sampling techniques.^17,18^ Using the approach of a PAS with a given path length, we have calculated this value experimentally, for the SPGE, obtaining an SR value of 0.091 m^3^ day^–1^, for 1 min of exposure by employing the right part of eq 9, that is, knowing the mass of the amalgamated mercury, the time of exposure, and the GEM concentration inside the jar. The SR value could also be calculated theoretically substituting the diffusion coefficient, the measured area of the surface, and the estimated diffusive path length for 1 min of exposure (Figure 5) in the left part of eq 9, which gives a value of 0.021 m^3^ day^–1^. Again, the difference between these SR values could be due to the value of the area. Our experimental SR values are similar to the reported data for passive samplers.^47^

### GEM Calibration

For the calibration of the combined GEM passive sampling and voltammetric detection system, volumes of 1.0, 2.4, 5.0, 7.6, and 10.0 cm^3^ of air were taken from the GEM stock bottle (Figure 2) and injected into a second bottle containing the SPGE (Figure 3) in separate experiments, giving final GEM concentrations in the range from 5.82 to 59.29 ng dm^–3^. Three SPGEs were exposed in each experiment (triplicate measurements). Sampling time was 30 min, as previously optimized.

**Figure 7:**
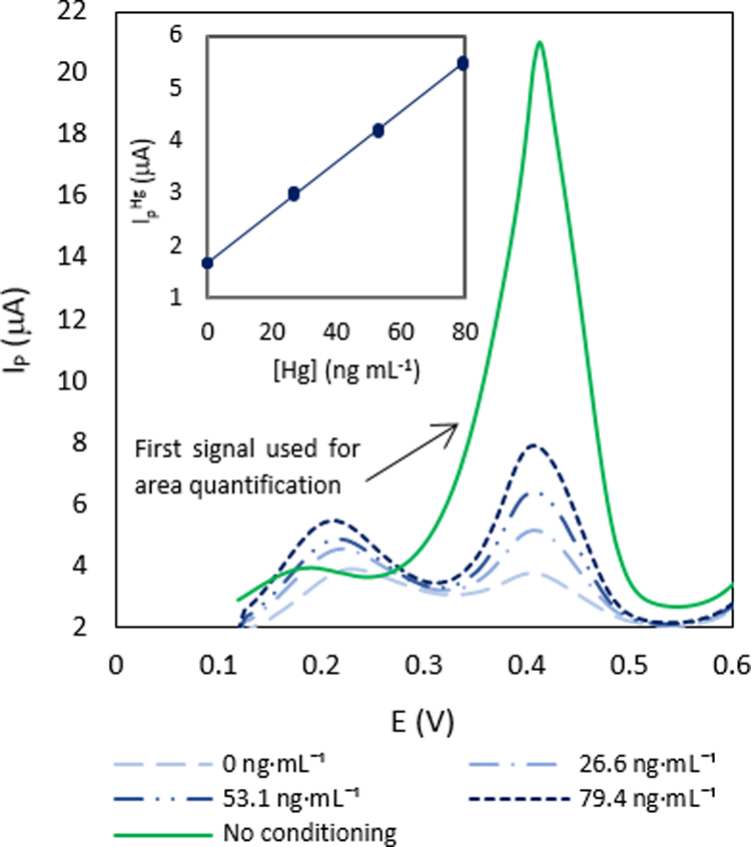
Solid green line: signal obtained for measurement protocol 1 (see Measurement Protocol 1 section). Blue lines: signals obtained and the calibration curve for 26.6–53.1 ng mL^–1^ Hg(II) standard additions for measurement protocol 2 (see Measurement Protocol 1 section). The LT-SPGE is exposed for 30 min to 56.59 ng dm^–3^ GEM.

**Figure 8:**
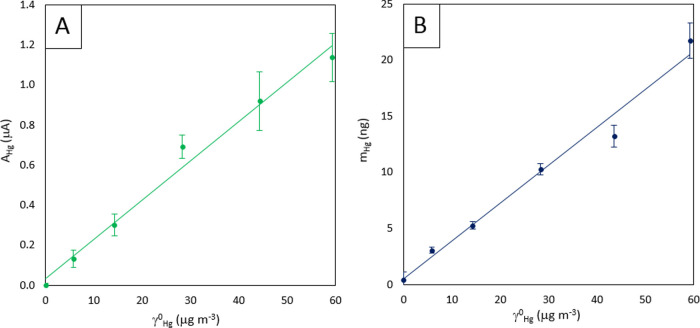
Calibration of 5.82–59.69 ng dm^–3^ GEM solutions on the LT-SPGE exposed for 30 min. (A) Analytical signal *A*_Hg_. (B) Analytical signal *m*_Hg_. Experimental conditions: deposition time: 60 s; deposition potential: −0.1 V; stirring rate: 300 rpm; cleaning step: 30 s at 0.7 V (only for *m*_Hg_); SWV settings: step potential, 6 mV; frequency, 10 Hz; amplitude, 40 mV; initial potential, 0.1 V; and final potential, 0.65 V.

As described in Voltammetric Analysis section, the signals employed for the calibration curve were the area under the current peak, *A*_Hg_, measured according to protocol 1 (Figure 7, solid green line) and the mass of mercury collected by the electrode, *m*_Hg_, as determined by SWASV (Figure 7, blue lines) following the experimental measurement protocol 2. The Hg(II) standard additions employed in this protocol were 26.6, 53.1, and 79.4 ng mL^–1^ (Figure 7, blue lines). Both signals (*A*_Hg_ and *m*_Hg_) were used to obtain GEM calibration curves in Figure 8.

Table 1 summarizes the main calibration parameters obtained using measurement protocols 1 (analytical signal, *A*_Hg_) and 2 (analytical signal, *m*_Hg_). As can be seen, the GEM detection limits, calculated according to Long and Winefordner, are 5.32 and 5.22 ng dm^–3^ for *A*_Hg_ and *m*_Hg_, respectively. These values are too high for the application of this methodology in the determination of GEM in regular outdoor or indoor air, but this is the first proof of the applicability of SPGEs for sampling and voltammetric detection of GEM.

**Table 1:** Calibration Data for the Determination of GEM on an LT-SPGE in 0.1 M HCl^a^

signal	*m*	*B*	*S*_m_	*S*_b_	*S*_*y*_/*X*	AS (ng dm^–3^)	*R*^2^	linearity (%)	LOD (ng dm^–3^)
*A*_Hg_^b^	0.020	0.033	0.001	0.033	0.089	4.56	0.9581	94.90	5.3260
*m*_Hg_^c^	0.337	0.477	0.013	0.438	1.188	3.53	0.9782	96.04	5.2234

a AS: analytical sensitivity, LOD: limit of detection, and *m*: sensitivity [μA(ng)/ng mL^–1^].

b Measurement Protocol 1.

c Measurement Protocol 2.

## Conclusions

Printed gold electrodes cured at low temperatures (LT-SPGE) have been demonstrated for the first time as useful PASs for GEM collection and subsequent voltammetric detection (SWASV) of the sampled mercury.

GEM generation is possible with the use of “bell jars” containing a small amount of mercury to establish the dynamic equilibrium with the surrounding atmosphere. Accurate volumes of this mercury can be taken from the jar and diluted adequately for calibration purposes with gas-tight syringes.

LT-SPGE microscopic structural properties make it more suitable for GEM capture compared to SPGE cured at high temperatures (HT-SPGE). These electrodes show excellent stability for repeated voltammetric measurements that allow the quantification of the mass of mercury by standard additions.

Two experimental measurement protocols are possible to carry out the calibration curves for GEM quantification and the mass of collected GEM time dependency over the time assessment. In the first one, the voltammetric peak area is used as a signal and, in the other one, the mass of collected mercury, *m*_Hg_.

For the concentrations and times studied, the adsorption of *m*_Hg_ follows a linear behavior until the sorbent becomes saturated, which is confirmed by the TOF-SIMS analysis and by the theoretical approach described in the present work. The theoretical value of the SR is according to those found in the literature.

A time of 30 min could be established as a good compromise between a short time of analysis and enough sensibility for the analysis of GEM concentrations between 5.82 and 59.69 ng dm^–3^ showing good correlation coefficients for the two protocols.

Work is in progress to validate the new analytical strategy for GEM in real atmospheric samples.

## Supporting Information Available

SEM and TOF-SIMS results of SPGEs; peak height stability for repeated SWASV measurements; relative intensity of Hg^+^ ion on the SPGE surface after exposure to 59.69 ng dm^-3^ GEM concentration and its intensity upon exposure for different times; and *m*_Hg_ adsorbed onto the LT-SPGE for increasing GEM concentrations (PDF)
