# Supplementary material for: Screen-Printed Gold Electrodes as Passive Samplers and Voltammetric Platforms for the Determination of Gaseous Elemental Mercury
Source: Anal Chem. 2021 Feb 1;93(6):3122–9. doi: 10.1021/acs.analchem.0c04347 (PMC8562869; doi:10.1021/acs.analchem.0c04347)
Supplement: Supplementary file 1 — ac0c04347_si_001.pdf [file ac0c04347_si_001.pdf]

Supporting Information

Screen-printed gold electrodes as passive samplers and voltammetric platforms for the determination of gaseous elemental mercury

Samuel Frutos-Puerto [1,*], Conrado Miró [2] and Eduardo Pinilla-Gil [3].

[1] Department of Analytical Chemistry, University of Extremadura, Av. de Elvas, s/n, 06006, Badajoz, Spain; samfrutosp@unex.es

[2] Department of Applied Physics, University of Extremadura, Av. de la Universidad, s/n, 10005, Cáceres, Spain; cmiro@unex.es

[3] Department of Analytical Chemistry, University of Extremadura, Av. de Elvas, s/n, 06006, Badajoz, Spain; epinilla@unex.es

* Correspondence: samfrutosp@unex.es; Tel.: +34-924-289-389

Table of contents

Figure	Description
S1	SEM images for A) commercial LT-SPGE (DropSens 220BT), B) commercial HT-SPGE (DropSens 220AT) (adapted from García-González et al., 2008).
S2	TOF-SIMS ion distribution in a 500 x 500 micrometre region of DropSens 220BT SPGE after 30 minutes of exposition to an atmosphere containing a GEM concentration of 56.69 ng dm^{-3}. a) Au^+ ion, b) Hg^+, c) $AuHg^+$ and d) sum of ions.
S3	Relative intensity of Hg^+ ion on the SPGE surface after exposition to a 59.69 ng dm^{-3} GEM concentration.
S4	Intensity of Hg^+ ion on the working LT-SPGE surface after exposition to a 59.69 ng dm^{-3} GEM. A) 0, B) 60 and C) 120 minutes.
S5	Peak height stability for repeated SWASV measurement of 30 ng mL^{-1} of Hg(II) in 0.1 M HCl. Frequency 10 Hz, step potential 6 mV, amplitude 40 mV, deposition potential -0.1 V, stirring rate 300 rpm, and deposition time 60s.
S6	m_{Hg} adsorbed on the LT-SPGE over the time for GEM concentrations of 5.66, 28.30 and 56.69 ng dm^{-3}. Each point of the graph is determined by following the experimental measurement protocol 2 (see section 2).Experimental conditions: deposition time: 60 s; deposition potential: -0.1 V; stirring rate: 300 r.p.m.; cleaning step: 30 s at 0.7 V; SWV settings: step potential: 6 mV; frequency: 10 Hz; amplitude 40 mV; initial potential: 0.1 V and final potential 0.65 V.

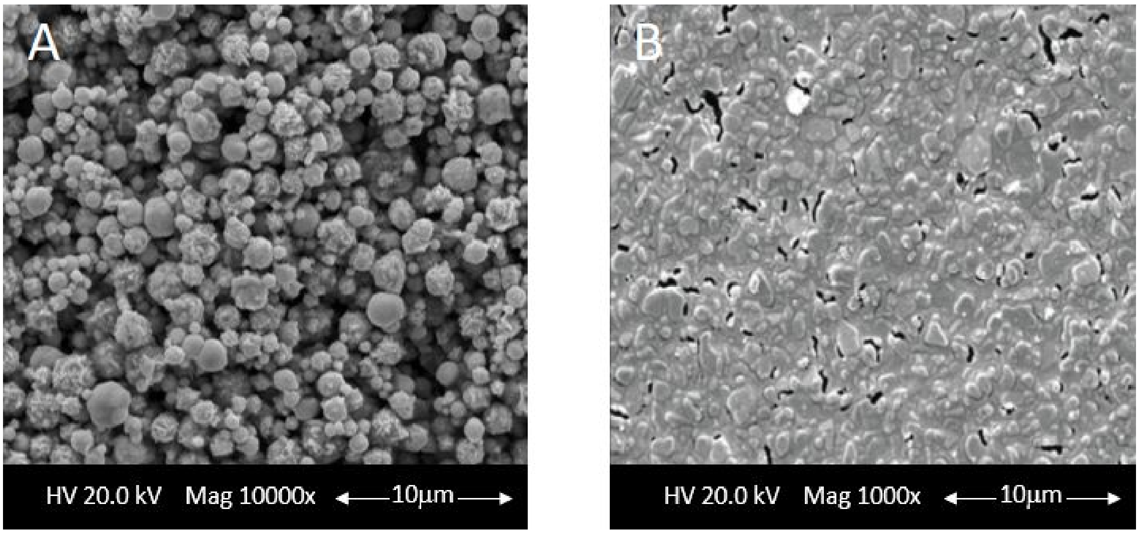

Figure S1. SEM images for A) commercial LT-SPGE (DropSens 220BT), B) commercial HT-SPGE (DropSens 220AT (adapted from García-González et al., 2008).

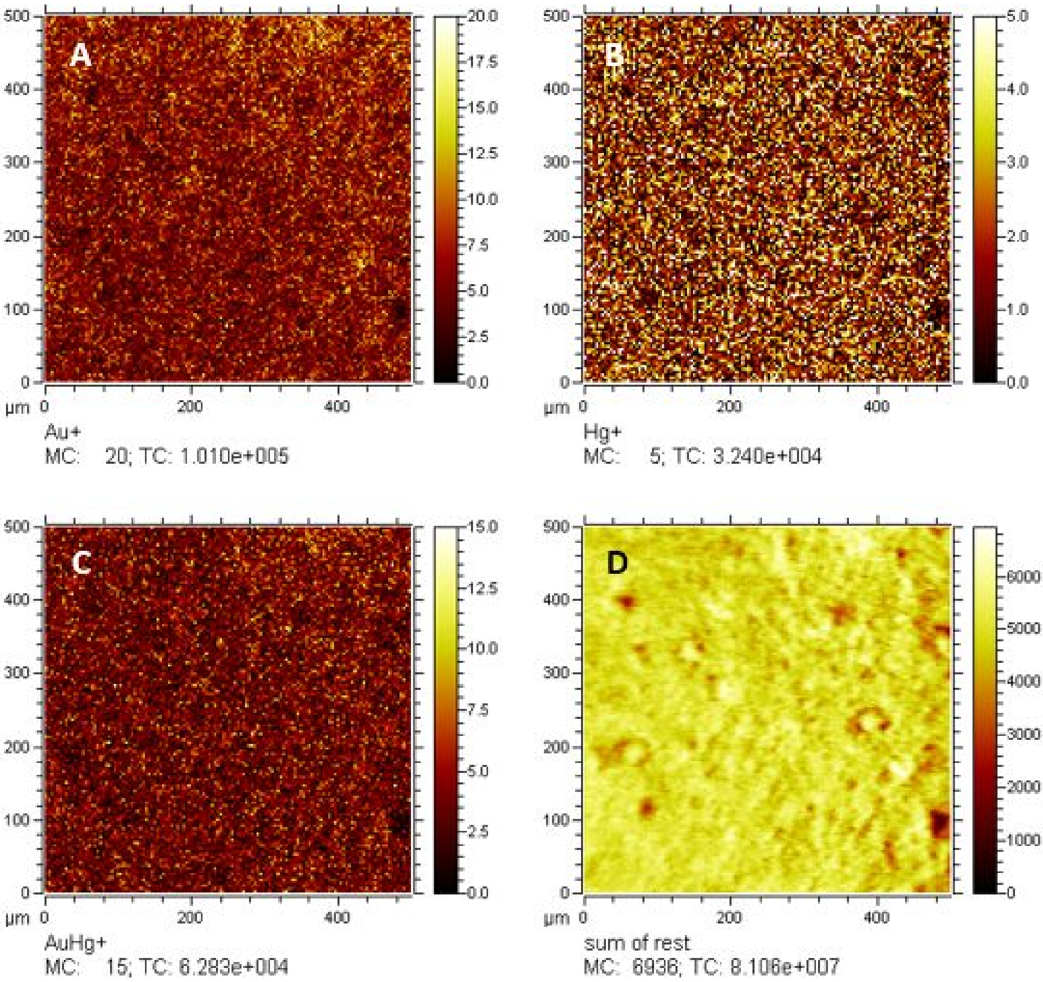

Figure S2. TOF-SIMS ion distribution in a 500 x 500 micrometre region of DropSens 220BT SPGE after 30 minutes of exposition to an atmosphere containing a GEM concentration of 56.69 ng dm^{-3}. a) Au^{+} ion, b) Hg^{+}, c) $AuHg^{+}$ and d) sum of ions.

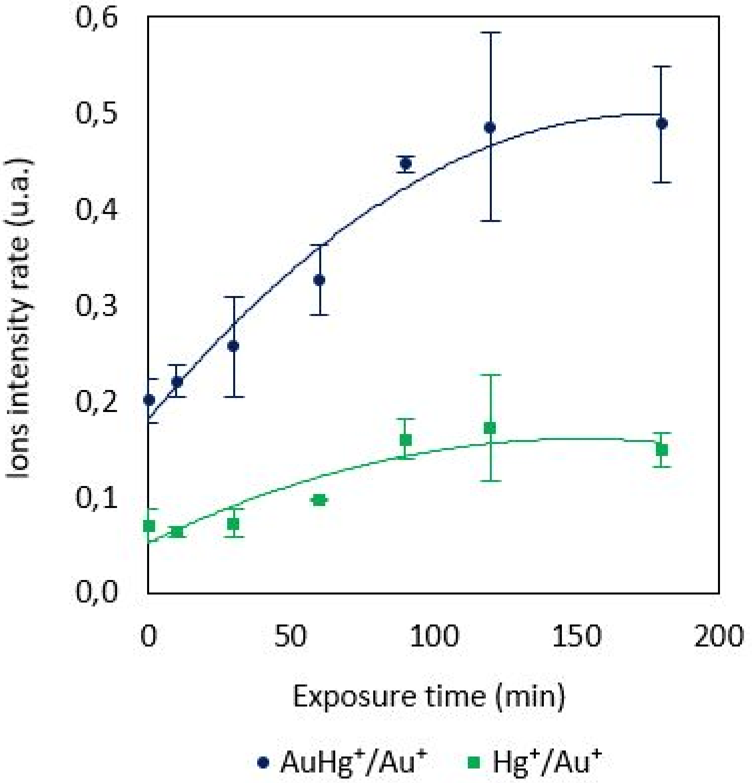

Figure S3. Relative intensity of Hg^+ ion on the SPGE surface after exposition to a 59.69 ng dm^{-3} GEM concentration.

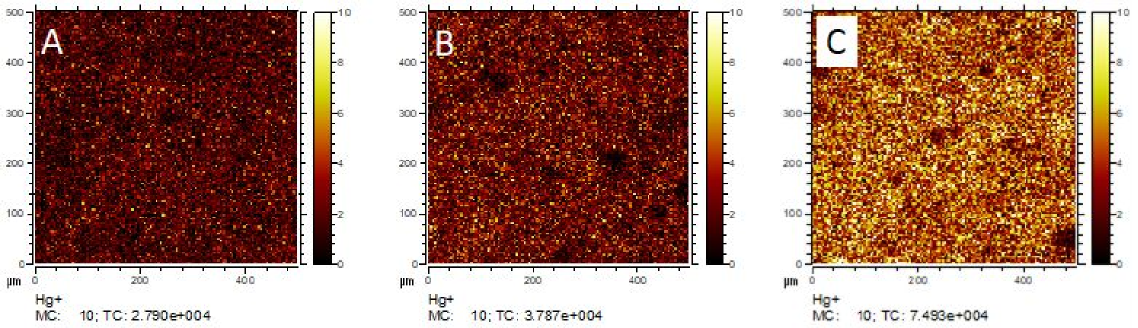

Figure S4. Intensity of Hg^+ ion on the working LT-SPGE surface after exposition to a 59.69 ng dm^{-3} GEM. A) 0, B) 60 and C) 120 minutes.

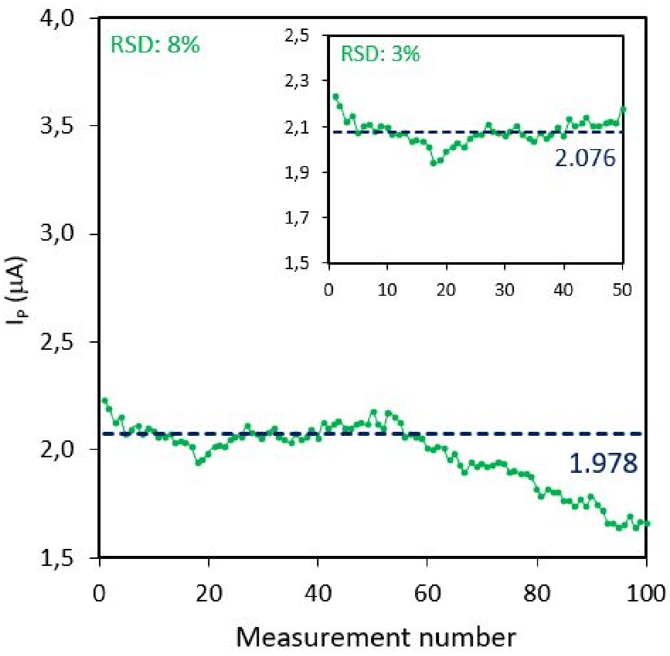

Figure S5. Peak height stability for repeated SWASV measurement of 30 ng mL^{-1} of Hg(II) in 0.1 M HCl. Frequency 10 Hz, step potential 6 mV, amplitude 40 mV, deposition potential -0.1 V, stirring rate 300 rpm, and deposition time 60s.

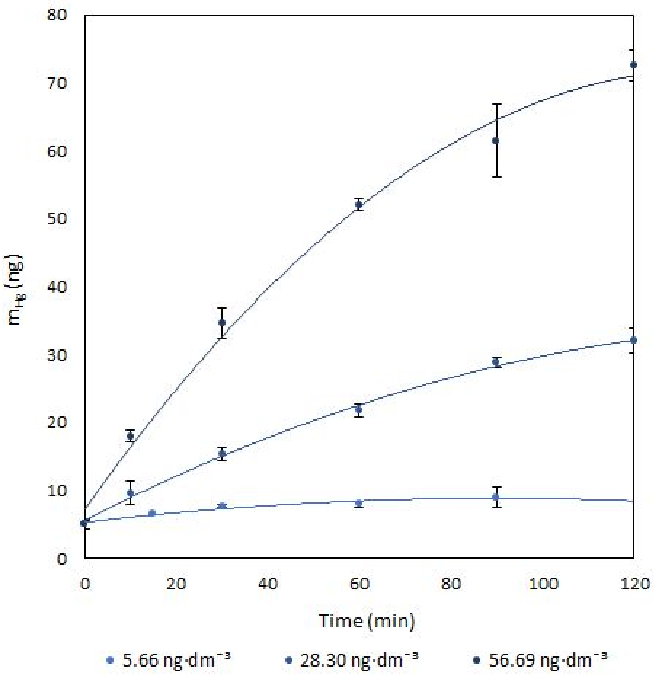

Figure S6. m_{Hg} adsorbed on the LT-SPGE over the time for GEM concentrations of 5.66, 28.30 and 56.69 ng dm^{-3}. Each point of the graph is determined by following the experimental measurement protocol 2 (see section 2).Experimental conditions: deposition time: 60 s; deposition potential: -0.1 V; stirring rate: 300 r.p.m.; cleaning step: 30 s at 0.7 V; SWV settings: step potential: 6 mV; frequency: 10 Hz; amplitude 40 mV; initial potential: 0.1 V and final potential 0.65 V.
